# Adaptation to Stress Switches Stress-Evoked Network Disconnection to a New Forebrain Network State

**DOI:** 10.1523/ENEURO.0336-25.2026

**Published:** 2026-08-19

**Authors:** Laura Durieux, Romain Bourdy, Karin Herbeaux, Nelson K. Totah, Lucas Lecourtier

**Affiliations:** ^1^Université de Strasbourg, CNRS, Laboratoire de Neurosciences Cognitives et Adaptatives (LNCA), UMR 7364, Strasbourg F-67000, France; ^2^Helsinki Institute of Life Science, University of Helsinki, Helsinki 00014, Finland; ^3^Faculty of Pharmacy, University of Helsinki, Helsinki 00014, Finland; ^4^Neuroscience Center, University of Helsinki, Helsinki 00014, Finland

**Keywords:** causality, coherence, gamma, local field potentials, stress adaptation, stress response, theta

## Abstract

Stress adaptation is critical to maintaining mental and physical health. If acute stress is associated with changes in neuronal activity in many brain regions, how neuronal activity itself is coordinated across regions during stress and how neuronal networks are modified during stress adaptation are unknown. In male rats, we recorded local field potentials (LFPs) from stress-responsive regions, including the prelimbic cortex, anterior cingulate cortex, basolateral amygdala, lateral habenula, and dorsal hippocampus, in rats during repeated stress exposure in a within-subjects design. We characterized network activity by computing cross-region coherence, Granger causality, and phase–amplitude coupling on bipolar derivatives of LFPs. We established a stress adaptation model in rats: the behavioral response to acute 10 min restraint stress returned to baseline after a second restraint 3 h later. The pre-stress state was characterized by robust global network interactions in the theta and gamma bands. The first exposure induced widespread disconnection, whereas following the second one, when rats showed an adaptive response, the connectivity pattern appeared different from that of the pre-stress state. Finally, baseline, stressed, and adaptive states were predicted (>90% accuracy) from network activity. Overall, we showed that the acutely stressed brain state was primarily a state of network disconnection, while stress adaptation was a new network state rather than a return to baseline.

## Significance Statement

Upon repeated stress exposure, individuals can adapt and cope. Such responses can be observed at the cerebral level through investigation of communication among a network of key regions involved in the stress response, using local field potential recordings and coherence and causality analyses. Here we demonstrate that rats exposed to two consecutive stressful experiences show behavioral signs of adaptation during the second and that stress and adaptive states are associated with distinct network activity; while the stress state is accompanied by widespread disconnections among the network, the adaptive state is associated with a distinct network configuration compared with the pre-stress baseline. Network communication could therefore hold translational potential for understanding stress and adaptive states and help delineate stress-related disorders.

## Introduction

Exposure to stressful events is an important feature of life, and being able to adapt to it is crucial for one's well-being. Adaptation to stress can be seen as the capacity to overcome adversity and threats, to maintain stability in everyday life. Adaptation, which promotes resilience, is orchestrated by physiological responses, such as those of the hypothalamic–pituitary–adrenal axis, engaged to maintain homeostasis, and results in the initiation of a tempered behavioral response, i.e., that resembles, in magnitude and duration, that displayed in the absence of stress exposure (Reul et al., 2015; Kinlein and Karatsoreos, 2020). On the contrary, maladaptation can lead to depression, anxiety, and posttraumatic stress disorder (Chrousos, 2009; McEwen et al., 2015; Godoy et al., 2018; Nestler and Russo, 2024). It is therefore of great importance to understand how the brain responds to stressful situations and how it adapts to repeated stress exposure. Encountering stressful situations and adapting to them alters the activity of numerous brain regions including the hypothalamic–pituitary–adrenal axis (de Kloet, 1992; de Kloet et al., 1999), the noradrenergic system (Pace and Myers, 2023), the medial prefrontal cortex (mPFC; McKlveen et al., 2015), the hippocampus (HPC; Suri and Vaidya, 2015), and the amygdala (AMY; Sharp, 2017). Nonetheless, considerably less is known about the cross-region coordination of neuronal activity during stress and during adaptation. Prior studies have used Fos protein expression to resolve the network activity patterns in the context of stress (Knox et al., 2016; Vetere et al., 2017; Careaga et al., 2019; Durieux et al., 2020). However, Fos is an indirect measure of neuronal activity and is not expressed by all cell types (Salery et al., 2021; Cruz-Mendoza et al., 2022). Most critically, Fos is a postmortem measure relaying only a static snapshot rather than tracking the same network throughout stress and adaptation. The local field potential (LFP), which is the superposition of the transmembrane currents produced (primarily) by synaptic inputs in the vicinity of the electrode, is ideal for inferring cross-region synaptic drive to define neuronal networks based on the underlying regional neuronal activity (Fries, 2005; Buzsáki et al., 2012). Moreover, such technique allows, through multiple recording epochs, to explore in the same subject the evolution of the network response to successive experiences, capturing acute stress response and subsequent adaptation. Studies addressed large network activity during and with regard to stressors (Seidenbecher et al., 2003; Adhikari et al., 2010; Stujenske et al., 2014; Hultman et al., 2016, 2018; Carlson et al., 2017; Abe et al., 2019; Nakayama et al., 2019), including, but rarely, during adaptation facing its repetition (Carlson et al., 2017), mainly showing stress-related increased network connectivity.

Here, we investigated in male rats the cross-region coordination of neuronal activity among a cortical-subcortical network of structures that has been shown to be correlatively engaged during various stress paradigms (Jacinto et al., 2013; Durieux et al., 2020, 2022; Merino et al., 2021; Marques et al., 2022), including the prelimbic cortex (PRL), anterior cingulate cortex (ACC), basolateral AMY (BLA), lateral habenula (LHb), and dorsal HPC (dHPC). We used a within-subject design in the context of repeated stress exposures (two 10 min restraint sessions 3 h apart). This exact stress type and duration with a 3 h delay has been used to show adaptive mesocortical dopamine release to the second stressor (Jackson and Moghaddam, 2004). Activation of this neuromodulatory pathway has been implicated in both the acute and adaptive stress responses (Cools and Robbins, 2004; Douma and de Kloet, 2020; Arnsten et al., 2023). We recorded multielectrode LFPs within each of the five regions, simultaneously, and used local bipolar derivations in each region to avoid volume conduction. We used a variety of behavioral measures to demonstrate adaptation after the second stressor. We characterized how cross-region neuronal interactions change in response to stress and during adaptation, focusing on the first minute of the wake period immediately following restraint, when attention and information processing are at the highest following an emotionally salient event (Karakaş, 2020; Okonogi and Sasaki, 2021; Fries, 2023). We computed cross-region coherence, Granger causality, and phase–amplitude coupling in the theta band and gamma band because stressful events in rodents are known to modulate these bands in stress-related forebrain networks (Seidenbecher et al., 2003; Adhikari et al., 2010; Colgin, 2013; Headley and Paré, 2013; Likhtik et al., 2014).

## Materials and Methods

### Animals

Experimental protocols and animal care were in compliance with the institutional guidelines (Council Directive 87/848, October 19, 1987, Ministère de l′Agriculture et de la Forêt, Service Veterinaires de la Santé et de la Protection Animale) and international laws (Directive 2010/63/UE, February 13, 2013, European Community) and policies: APAFIS#7114. The study required 15 male Long–Evans rats (250–350 g; Janvier Labs). Rats were pair housed (until surgery) on a 12 h light cycle (lights on—ZT0—at 07:00) at 22 ± 1°C (∼55% RH) with unrestricted food and water access.

### Surgery

An analgesic (meloxicam; 1 mg/kg, s.c.) was administered prior to anesthesia induction (isoflurane; 4% in an induction box for 3 min; 1.5% for maintenance). A lidocaine/bupivacaine mix (2 mg/kg each, s.c.) was injected for local analgesia at the incision site. A bundle of three electrodes (0.004″ PFA-coated tungsten, A-M Systems) was implanted in the right hemisphere in each region; the distance between the electrodes was ∼150 µm. Stereotaxic coordinates (mm) anteroposterior (AP) from bregma, mediolateral (ML) from the midline of the sagittal sinus, and dorsoventral (DV) from the dura were as follows: AP = +2.6, ML = 0.7, DV = −3 (PRL); AP = +0.5, ML = 0.7, DV = −2 (ACC); AP = −2.5, ML = 4.7, DV = −6.8 (BLA); AP = −3.5, ML = 0.7, DV = −4.4 (LHb); and AP = −3.8, ML = 2.7, DV = −2.1 (CA1 region of the dHPC). A 2% DiI solution (Sigma-Aldrich) was applied at the tip of each electrode for postmortem histological verification of their position. A reference screw was placed over the left frontal cortex, and a ground screw was placed over the cerebellum. Once all electrodes were positioned, they were glued (C&B, Super-Bond) to the skull and skull screws that provided structural support. Wires were soldered to vias of an electrode interface board (EIB-16, NeuraLynx). The wound margin was filled in with dental cement. Rats were placed in clean cages under a heating lamp until complete awakening. On the day following the surgery, rats received a second injection of meloxicam (1 mg/kg, s.c.). Following surgery, rats remained single housed. Experiments began following a 10 d recovery period during which the well-being of the rats was daily checked.

### Stress paradigm and behavioral scoring

**Figure 1.**
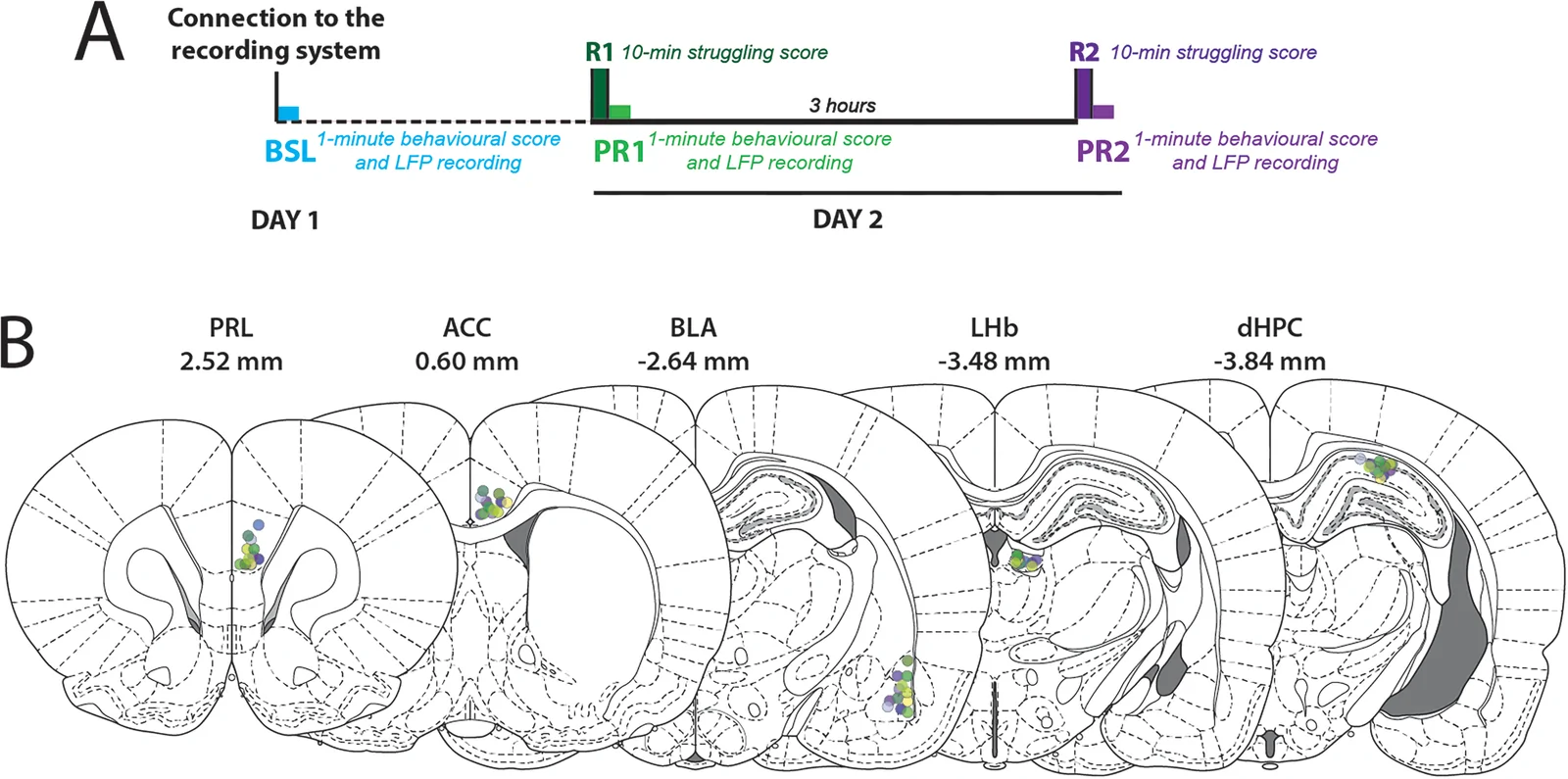
Recording of LFPs from five forebrain regions simultaneously during a repeated restraint stress paradigm. ***A***, Timeline of the experiment. On DAY 1, we made a 1 min baseline LFP recording and quantification of behavior. The stress exposure experiment took place on the following day (DAY 2). It consisted of two 10 min restraint stressors [restraint 1 (R1) and restraint 2 (R2)], 3 h apart, during which the time spent struggling was recorded. Behavioral activity was scored and LFP recorded for 1 min during BSL immediately following connection to the system and immediately following each stressor; these are referred to as the post-restraint 1 (PR1) and post-restraint 2 (PR2) epochs of the experiment. ***B***, Postmortem histology was used to determine the locations of the tip of the three-electrode bundles implanted within the PRL, ACC, BLA, LHb, and the CA1 region of the dHPC. The site for each rat (*n* = 10) is shown in a different color. The numbers above each coronal brain diagram show the distance from bregma in the anteroposterior axis.

First, over 3 consecutive days, for 3 h/d, rats were habituated to being connected to the rotating commutator and to being moved to a different room and placed within a faraday cage; on the third day (designated as DAY 1 in Fig. 1*A*), neuronal activity was recorded as a baseline (BSL). On the following day, rats were exposed to repeated stress exposure consisting of two 10 min restraint sessions performed 3 h apart. Rats were connected to the recording system for 1 h prior to the first restraint. Recordings were performed during 1 min following each restraint. Restraint sessions had to be performed between 09:00 and 13:00 when CORT secretion, an indicator of the activity of the stress response axis, is minimal and stable (Kalsbeek et al., 1996; Atkinson and Waddell, 1997). Therefore, BSL measures were acquired the previous day to avoid the study, would had it been performed on a single day, to span too much throughout the light period; in that case, the second restraint would have occurred too close to the rise of diurnal CORT secretion, with the potential risk of inducing circadian-based biases. The entire procedure was performed in the rat's home cage. During both restraints, the time spent struggling was manually scored by the person doing the restraint. During BSL, following connection to the system, and following each restraint session, behaviors were observed and manually timed from the videos for 1 min (Fig. 1*A*). These behaviors included some often associated with the stress response, such as self-grooming (van Erp et al., 1994; see review Li et al., 2024) and locomotion (Berridge and Dunn, 1987), although others found no change or a decrease following restraint (Klenerová et al., 2007). They also included peculiar behaviors, often associated with response to psychoactive drugs, but that can be considered part of a generalized stress-induced behavioral activation, including sniffing down, in which rats are positioned head down with their nose below the horizontal plane of their body while either standing or walking (Cole and Koob, 1989), digging that consists of spreading of bedding material with the forepaws (Diamant and de Wied, 1991; Tyler et al., 2020), wet-dog shake that consists of a paroxystic shudder of the head, neck, and trunk (Bedard and Pycock, 1977), and circling that consists of making a turn (Costall and Naylor, 1974). To summarize behavioral measures and reduce dimensionality, principal component analyses (PCAs) were performed separately for each epoch using the scored behavioral data. Components with eigenvalues >1 (Kaiser criterion) were kept, and variables with loadings ≥0.6 were considered substantial contributors to components.

### Electrophysiology signal processing and analyses

#### Signal acquisition

The broadband (0.5 Hz to 8 kHz) signal was sampled at 1,375 Hz (AlphaLab amplifier, Alpha Omega; and a preamplifier from ADInstruments). Electrode impedance at 1 kHz was ∼50–200 kΩ.

#### Preprocessing

Analyses were done in MATLAB R2020b (MathWorks). Signals were downsampled to 275 Hz and then bandpass filtered (0.5–100 Hz) using a second-order Butterworth acausal filter. To avoid contamination of the local signal by volume-conducted signals from surrounding regions, for each region, we calculated bipolar derivations by subtracting the signals recorded on two of the three channels. We chose the two electrodes with minimal artifactual contamination throughout the experiment. We then removed slow drift using the locdetrend function from the Chronux toolbox (http://chronux.org/). Movement artifacts were removed using an artifact detection and removal algorithm based on stationary wavelet transform (Islam et al., 2014). Signals were visually inspected to ensure that artifacts and clipped signals were excluded from analysis.

#### Validation of wakefulness

We confirmed that rats were awake by checking for wake-associated field potentials and the presence of body movement. Movement was measured by automated, markerless tracking of the rat's head from the video recordings using DeepLabCut (DLC; 2,3). Video was recorded (30 fps). Training DLC included 11 videos with 100 images and was created with the pretrained network ResNet152. We used imaug (https://github.com/aleju/imgaug-doc) as an image augmenter and filtered the data using the median filter to remove outliers. We checked for wake-associated field potentials, which are characterized by a high theta/delta power ratio in dHPC and low delta/gamma power ratio in PRL (Gervasoni et al., 2004). The analyses were done on the bipolar derivatives of the LFP.

#### Spectral analyses

All analyses used the Chronux toolbox. Power was calculated using a 4 s sliding window in 0.2 s steps using the function mtspecgramc. We used nine tapers (*k* = 9 in Chronux), and the time-half bandwidth product (nw) was set to five. Power was *Z*-scored, within each frequency bin, to the time-binned power of the recorded signal. Coherence was calculated for each cross-region recording site pair in each epoch (BSL, PR1, PR2) using the function cohgramc. We assessed whether coherence values were significant using a one-sided permutation test. A distribution of 1,000 chance coherence values was generated by bootstrapping. Surrogate data were made by cutting the signal at a random time point and flipping the two signal segments in time (Canolty et al., 2006) and repeating this process 1,000 times. This yielded 1,000 coherence values. If the coherence in the real data was higher than the top 1% of the 1,000 surrogate values, then it was considered a significant coherence value at *p* < 0.01. We used the method of Canolty et al. (2006) because it responds to the 1/f structure of the power spectrum and the voltage distribution of the signal, while only altering its temporal relation between the signals at each recording site.

To assess the relationship between behavioral responses and network activity, Pearson’s correlation coefficients (*r*) were computed between behavioral PCA scores and coherence values, for pairs showing significant coherence, separately for each epoch (BSL, PR1, PR2) and frequency band (theta, gamma). Statistical significance was assessed using a bootstrap procedure (1,000 resamples) to estimate 95% confidence intervals of the correlation coefficients. Correlations were considered significant when the confidence interval did not include zero.

#### Graph theory analyses

Graphs consisted of recording sites (one per brain region) as nodes and links between regions. A link was drawn by thresholding based on significant recording site-pair coherence in at least 80% of the rats.

#### Multivariate Granger causality calculation

We used the MVGC toolbox (Barnett and Seth, 2014) to calculate Granger causality using the standard parameters for MVGC. Pairwise Granger causality magnitude was conditioned upon one of the remaining recording sites and calculated once for each of the remaining sites. These conditioned magnitudes were then averaged to reduce spurious results due to global network activity. Statistical significance was assessed using a permutation test (×1,000, *p* < 0.01).

#### Phase–amplitude coupling analysis

We calculated the modulation index (MI) to quantify the degree to which the amplitude of gamma band oscillations is modulated by the phase of theta band oscillations. We used the method of Tort and colleagues (Tort et al., 2010b). The calculation was performed between recording sites that exhibited significant Granger causality. Following this method, we used surrogate data (see above, Spectral analyses) to compute a surrogate distribution of MI values and subtract the threshold value at which the MI was significant for each phase–amplitude bin. As a result, any MI values reported in the paper are significant if they are >0. For each of the 10 subjects, MI was calculated for each phase–amplitude bin separately for each pair of regions in each epoch (BSL, PR1, PR2). The across-subject average is presented as a co-modulogram.

#### Assessment of the generalization of the feedforward neuronal network: deep learning classifier

We used a built-in classifier from MATLAB (ClassificationNeuronalNetwork) to predict experimental epochs from coherence values calculated in 2 s bins during the first minute of the awake state for each epoch; 834 and 672 observations were used to train the classifier, and the remaining 20% were used as test data. We computed the algorithm with a Bayesian optimization to obtain the best neural network possible based on our dataset (Extended Data Fig. 6-2). The fitcnet function was used to create the neural network classifier. The classifier was trained using a feedforward algorithm with a binary cross-entropy loss function. Accuracy was determined based on the number of times the prediction was correct across the total number of predictions. We evaluated the prediction accuracy using a surrogate distribution (×1,000) generated by shuffling the epoch label. We assessed generalization to external data to avoid overtraining by holding-out data from one randomly selected rat from the training dataset and subsequently testing with held-out data (Extended Data Fig. 6-3).

### Histological verification of electrode placements

Rats were deeply anesthetized (pentobarbital, 120 mg/kg, i.p.). We performed intracardiac perfusion with 0.1 M PBS followed by 4% paraformaldehyde (PFA; pH 7.4; 4°C). Brains were removed, postfixed in 4% PFA (4°C) for 4 h, and then transferred into a 20% sucrose solution (in 0.1 M PBS) at 4°C for 48 h. Brains were frozen by immersion in isopentane (−35°C) for 1 min. Serial 40-μm-thick coronal sections were collected and mounted on gelatin-coated slides with a DAPI-Fluoromount medium. DiI was visualized (Apotome.2, Zeiss) to determine the location of the recording sites.

### Statistical analyses

Only rats (*n* = 10) with correct electrode placement in all five regions of interest and with minimal signal artifacts during the entire experiment on at least two of the three electrodes were used for analyses. We compared behavioral and neurophysiological measures between BSL, PR1, and PR2 epochs using within-subjects statistical tests. We verified the normality of the data and used a repeated-measures ANOVA with Newman–Keuls post hoc tests. The data for struggling were not normally distributed, and we compared PR1 to PR2 using the nonparametric Wilcoxon test. Coherence, modulation index, and Granger causality magnitude values that do not indicate coupling will still result in a calculated value. We assessed whether these values were significant using a permutation test which is described above.

## Results

We aimed to characterize neuronal activity coordination across multiple stress-responsive regions, during the acute response to stress and during adaptation to a repeated stressor. We used a within-subjects, longitudinal design. All data (behavioral and neurophysiological) were collected from 10 rats. We performed a repeated stress procedure consisting of two 10 min restraints 3 h apart, preceded by a baseline (BSL) the day before (Fig. 1*A*). Rats were habituated to the connection of the recording cable for multiple days prior to the BSL session. Behavior was scored and LFPs were analyzed in the first minute of BSL or the first minute after the first or second restraint stress (PR1 or PR2, respectively). Our goal was to infer synaptic drive between nodes in a network of brain regions. LFPs are the superposition of the transmembrane currents produced (primarily) by synaptic inputs in the vicinity of the electrode, which makes the LFPs ideal for inferring synaptic drive (Logothetis, 2003; Fries, 2005; Buzsáki et al., 2012). However, LFPs can be volume conducted away from the current source. Therefore, we calculated bipolar derivations from two electrodes in each of the five stress-responsive brain regions. These five brain regions were recorded simultaneously (Fig. 1*B*).

### Rats exhibit behavioral signs of adaptation to stress

**Figure 2.**
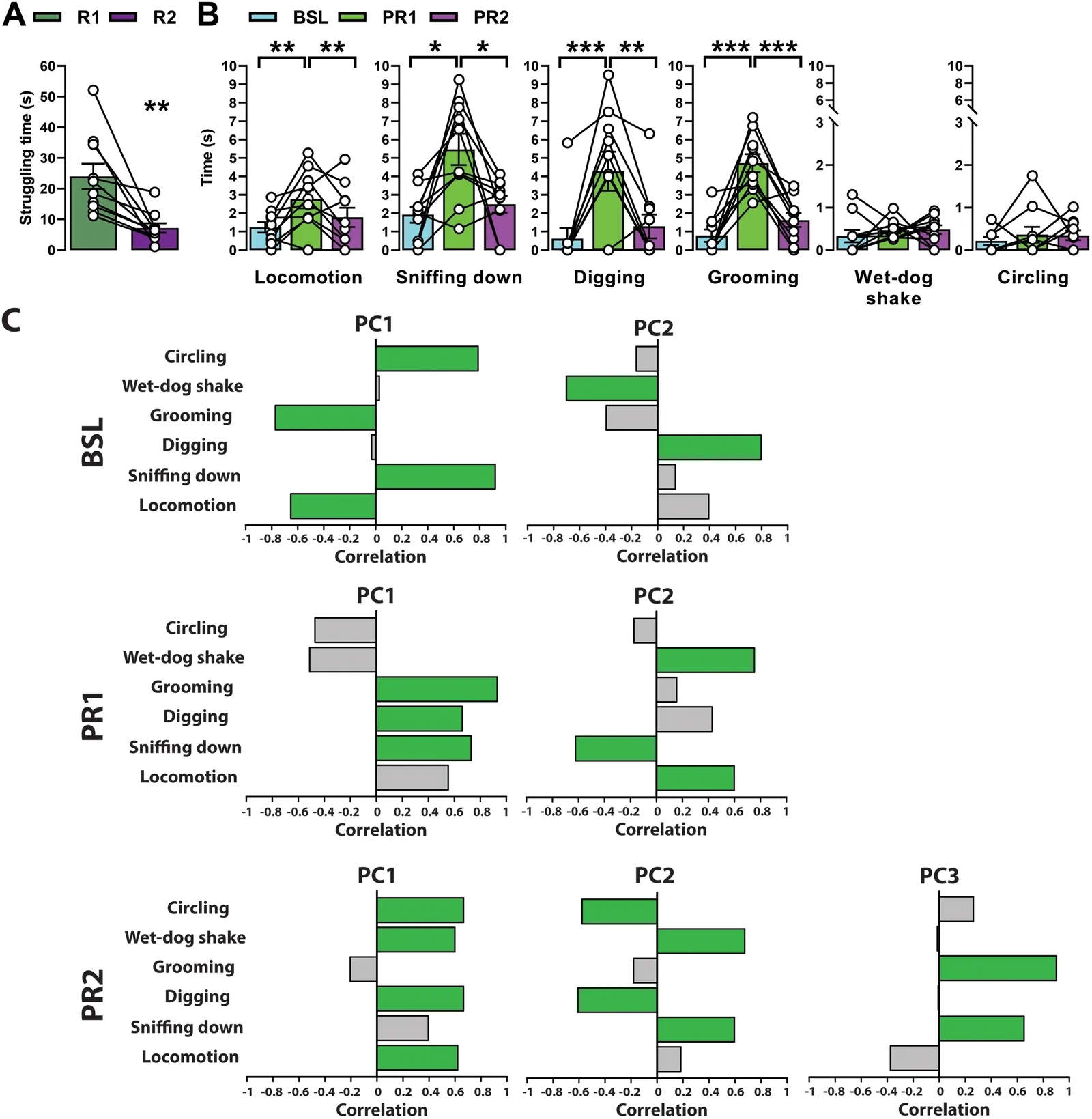
Scoring of behavioral response. Behavioral scoring (mean ± SEM; white circles represent individual data) indicated signs of adaptation during and following the second restraint. ***A***, Struggling time during the first (R1) and second (R2) restraint. ***B***, Time spent displaying each of the quantified behaviors during the 1 min scoring during baseline (BSL), post-restraint 1 (PR1), and post-restraint 2 (PR2). ***C***, Principal components (PCs) kept following individual PCA according to BSL (top), PR1 (middle), and PR2 (bottom). In green are behaviors with loadings ≥0.6 that were considered major contributors (detailed loadings are presented in Extended Data Fig. 2-1; results of a global PCA including the three epochs, including eigenvalues and explained variances, are presented in Extended Data Figs. 2-2 and 2-3). *p < 0.05, **p < 0.01, ***p < 0.001.

First, we needed to establish a behavioral paradigm in which rats would show stress adaptation. We defined adaptation as a “normalization” or “return-to-baseline” of a variety of behaviors that are known to be stress related, including struggling during restraint, and rats spent less time struggling during the second restraint compared with the first restraint (Wilcoxon signed-rank test, *W* = 2.80, *p* = 0.0051; Fig. 2*A*), as already demonstrated (Grissom et al., 2008), which indicates an adaptive response upon reexposure to the stressor. During the minute following each of the two restraint sessions, analysis of individual behaviors (Fig. 2*B*) indicated that the first restraint preferentially impacted locomotion (*F*_(2,18)_ = 5.88, *p* = 0.011; post hoc: BSL vs PR1, *p* = 0.009; PR1 vs PR2, *p* = 0.043; BSL vs PR2, *p* = 0.24), sniff down (*F*_(2,18)_ = 4.41, *p* = 0.028; post hoc: BSL vs PR1, *p* = 0.029; PR1 vs PR2, *p* = 0.038; BSL vs PR2, *p* = 0.57), digging (*F*_(2,18)_ = 11.51, *p* = 0.0006; post hoc: BSL vs PR1, *p* = 0.0009; PR1 vs PR2, *p* = 0.002; BSL vs PR2, *p* = 0.42), and grooming (*F*_(2,18)_ = 22.59, *p* = 0.00001; post hoc: BSL vs PR1, *p* = 0.0001; PR1 vs PR2, *p* = 0.0002; BSL vs PR2, *p* = 0.19). On the other hand, the first restraint affected neither wet-dog shake (*F*_(2,18)_ = 0.51, *p* = 0.61) nor circling (*F*_(2,18)_ = 0.34, *p* = 0.71). Altogether, these analyses suggest that the paradigm likely resulted in the succession of two distinct states. During PR1, the large increase in the time spent displaying stress-related behaviors indicates that this epoch represents a “stressed state.” This is supported by previous studies in rats showing the presence of physiological hallmarks of stress following restraint (Jackson and Moghaddam, 2004; Swanson et al., 2004; Mo et al., 2008; Ngampramuan et al., 2008; Durieux et al., 2022; Wongsaengchan et al., 2023). On the other hand, rats struggle less during the second restraint and spend less time displaying behavioral activation in its aftermath. Thus, this epoch represents an “adaptive state.” Assumptions regarding these behavioral states were further supported by PCA (Fig. 2*C*, Extended Data Fig. 2-1), which showed that PR1 and PR2 were characterized by distinct contributions of behavioral variables. In addition, differences were also observed between BSL and PR2, with PR2 displaying a less defined structure, as reflected by the keeping of three principal components. A global PCA across all sessions (Extended Data Figs. 2-2, 2-3) further confirmed the separation of behavioral states across conditions. Together, these results indicate that behavioral responses during the adaptive state differ from those observed at baseline, despite similar overall levels of behavioral activation.

10.1523/ENEURO.0336-25.2026.f2-1 Figure 2-1 Principal component (PC) analyses according to behavioral scoring during BSL, PR1 and PR2. Eigenvalues, explained variance (%), and cumulative variance of all PCs. According to BSL, two PCs were kept, explaining 66.18 % of the total variance. According to PR1, two PCs were kept, explaining 69.55 % of the total variance. According to PR2, three PCs were kept, explaining 80.81 % of the total variance. In green are PCs kept based on Eigenvalue > 1. Download Figure 2-1, TIF file.

10.1523/ENEURO.0336-25.2026.f2-2 Figure 2-2 Global principal component (PC) analyses according to behavioral scoring during the three epochs, showing eigenvalues and explained variance (%). In green are the two PCs kept, based on Eigenvalue > 1, explaining 56.26 % of the total variance. Download Figure 2-2, TIF file.

**Figure 3.**
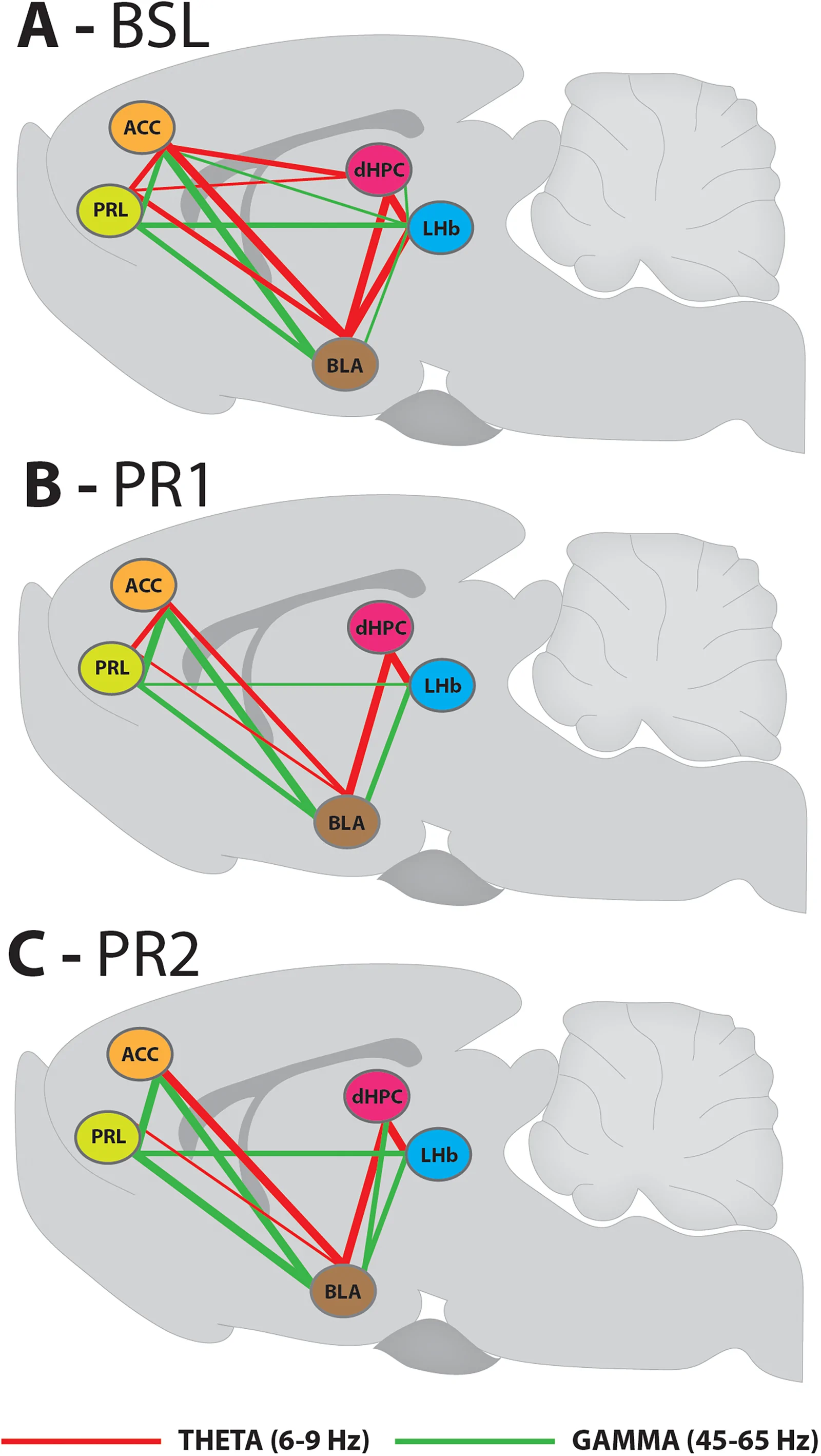
Stress and adaptive states are associated with both theta and gamma band network disconnection and reorganization, respectively. Graph networks depict significant cross-region coherence as thick lines in the theta (red) and gamma (green) bands during BSL (***A***), PR1 (***B***), and PR2 (***C***). Line thickness is related to coherence magnitude (small, coherence values <0.5; medium, coherence values between 0.5 and 0.6; large, coherence values >0.6). Subject-averaged power spectra are presented in Extended Data Figure 3-1, subject-averaged coherence spectra are presented in Extended Data Figures 3-2–3-4, correlation analyses between PCA scores and significant coherence values are presented in Extended Data Figure 3-5, and coherence values are presented in Extended Data Figure 3-6.

10.1523/ENEURO.0336-25.2026.f2-3 Figure 2-3 **A**) Principal components (PCs) kept following *global PCA* analyses based on eigenvalues > 1 (**Extended data Figure 2-2**). In green are behaviors with loadings ≥ 0.6 that were considered major contributors, for PC1 (*left*; locomotion, sniffing down, digging, grooming) and PC2 (*right*; wet-dog shake, circling). **B**) Scatter plot of individual values at each epoch based on the two dimensions kept from the global PCA, including confidence ellipses (95%) with centroids represented by larger symbols. Download Figure 2-3, TIF file.

### Stress decouples large-scale, global network activity patterns in the theta and gamma bands

We showed, during the first post-restraint minute, that not only the magnitude of the behavioral response varied in relation to stress and adaptive states across repeated stress exposures, in comparison with BSL. We selected this window because it captured the moment when the rat had most recently experienced stress and allows to address the pattern of these responses, as assessed by PCAs. Behavioral PCA revealed that responses following the two restraint sessions were characterized by distinct contributions of behavioral variables. Accordingly, PR1 and PR2 occupied different positions in the PCA space, indicating distinct behavioral profiles across conditions.

We first confirmed that all 10 rats were awake during this epoch by affirming that the bipolar derivatives of the LFP were in a wake-associated state and by observation of body movement (see Materials and Methods for details). We analyzed bipolar derivatives from two electrodes in each brain region to avoid volume conduction of the LFP across brain regions, and we refer to the bipolar derivative in each brain region as a “recording site.” Spectral power analysis of the bipolar derivative revealed theta and gamma band oscillations at all of the recording sites (subject-averaged power spectra in Extended Data Fig. 3-1). These bands have been previously associated with stress exposure in rodents (Seidenbecher et al., 2003; Adhikari et al., 2010; Colgin, 2013; Headley and Paré, 2013; Likhtik et al., 2014).

In order to study neuronal networks, we calculated coherence between recording sites. Coherence can be indicative of direct synaptic interactions between two areas or common synaptic input to the two areas. We found that coherence across all recording site pairs is primarily in the theta and/or gamma band (see subject-averaged coherence spectra in Extended Data Figs. 3-2–3-4). Notably, the pairwise coherence is different across recording site pairs, showing that the bipolar derivatives are not global and not volume conducted but are rather unique to each cross-region pair. We used graph theoretic measures to plot the topology of the stress network by drawing a link between recording sites if at least 80% of the rats had significant (permutation test; see Materials and Methods) theta or gamma coherence. We chose this approach of thresholding significant coherence values, as opposed to comparing coherence values in a repeated-measures ANOVA, because coherence values that are not indicative of any coupling at all will still have a value. Such a comparison would lead to a distorted view of network activity because it compares values that have no meaning. Our results show that, relative to BSL (Fig. 3*A*), acute stress exposure is associated with amygdalo-habenular and frontal-hippocampal disconnections in a network characterized by theta band oscillations (Fig. 3*B*); on the other hand, in the gamma band, the dHPC is completely segregated from the other four regions, while the LHb also becomes disconnected from the ACC (Fig. 3*B*). Overall, our results suggest that there is widespread coherence between all five regions during the BSL state and that the stressed state is primarily characterized by widespread disconnection of many nodes of this BSL state network.

10.1523/ENEURO.0336-25.2026.f3-1 Figure 3-1 Power spectrums of each brain region (LHb, ACC, dHPC, PRL, BLA) during the first AW min during BSL (grey), PR1 (blue), and PR2 (purple). Each power spectrum is separated into two parts according to low frequency band (1-10 Hz) with a linear scale for x axis, and high frequency band (20-80 Hz) with a logarithmic scale for x axis. Thick lines represent the standard deviation in the same color code than the given epoch. The orange and green vertical columns represent delimitations of the frequency bands of interest, theta range (6-9 Hz) and gamma range (45-65 Hz) respectively. Download Figure 3-1, TIF file.

10.1523/ENEURO.0336-25.2026.f3-2 Figure 3-2 Percentage of rats (left y-axis, blue bar plots) with significant coherence for each recording site-pair during baseline. The mean coherence (right y-axis, orange lines) is plotted across frequency (x-axis; logarithmic scale) between each of the 10 recording site-pairs. On each plot, the two vertical red lines frame the theta band (6-9 Hz), the two vertical green lines frame the gamma band (45-65 Hz), and the horizontal black line marks the 80 % value according to the number of rats showing significant coherence. Download Figure 3-2, TIF file.

10.1523/ENEURO.0336-25.2026.f3-3 Figure 3-3 Percentage of rats (left y-axis, blue bar plots) with significant coherence for each recording site-pair during PR1. The mean coherence (right y-axis, orange lines) is plotted across frequency (x-axis; logarithmic scale) between each of the 10 recording site-pairs. On each plot, the two vertical red lines frame the theta band (6-9 Hz), the two vertical green lines frame the gamma band (45-65 Hz), and the horizontal black line marks the 80 % value according to the number of rats showing significant coherence. Download Figure 3-3, TIF file.

**Figure 4.**
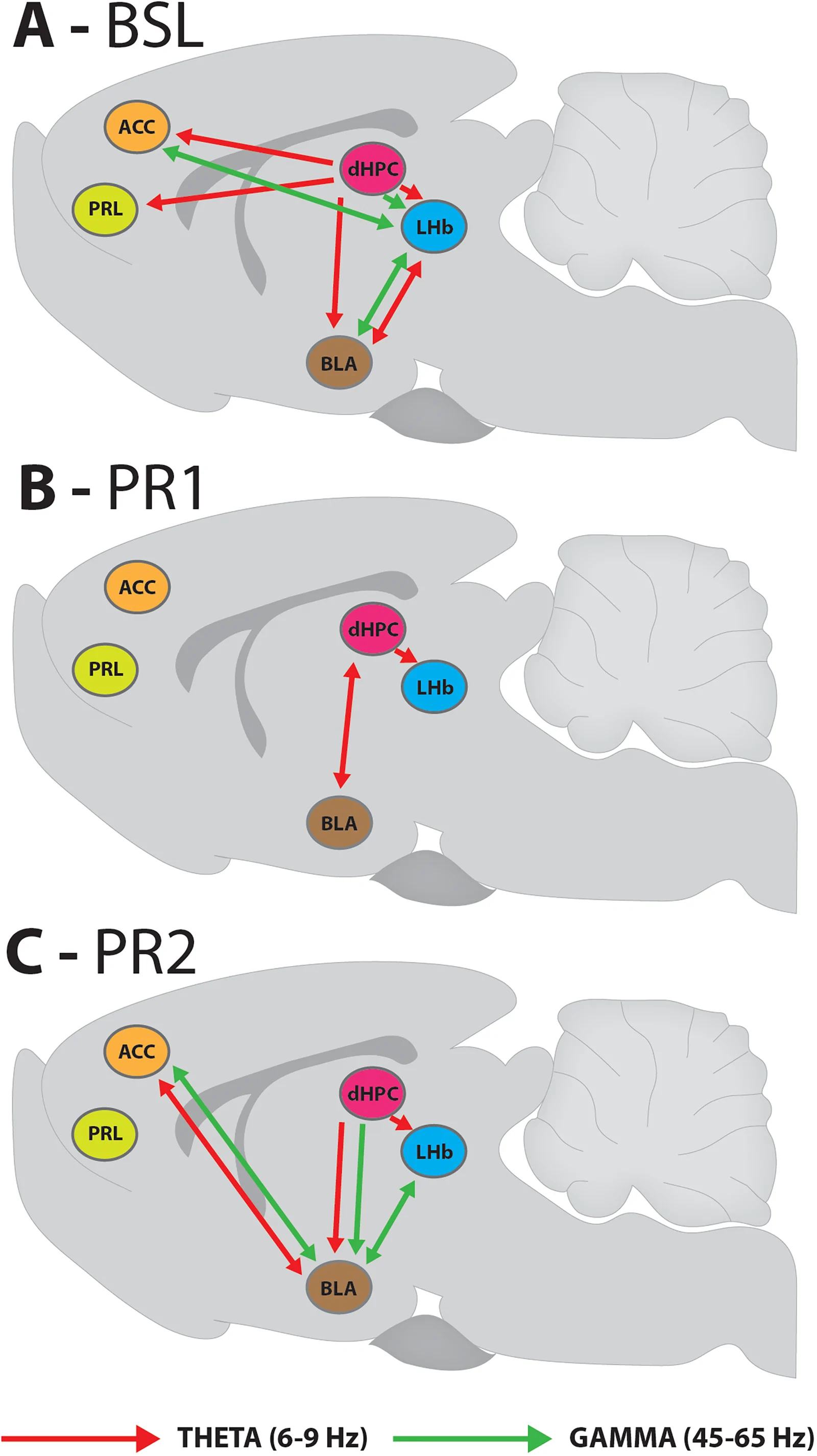
Granger causality analysis revealed a stress-associated state of network disconnection and an adaptive state characterized by a new network connectivity pattern. Graph networks depict relationships within the network in the theta (red) and gamma (green) bands during BSL (***A***), PR1 (***B***), and PR2 (***C***) [only significant relationships (unidirectional, single-headed arrows; bidirectional, double-headed arrows) are shown; values are presented in Extended Data Figs. 4-1, 4-2].

10.1523/ENEURO.0336-25.2026.f3-4 Figure 3-4 Percentage of rats (left y-axis, blue bar plots) with significant coherence for each recording site-pair during PR2. The mean coherence (right y-axis, orange lines) is plotted across frequency (x-axis; logarithmic scale) between each of the 10 recording site-pairs. On each plot, the two vertical red lines frame the theta band (6-9 Hz), the two vertical green lines frame the gamma band (45-65 Hz), and the horizontal black line marks the 80 % value according to the number of rats showing significant coherence. Download Figure 3-4, TIF file.

**Figure 5.**
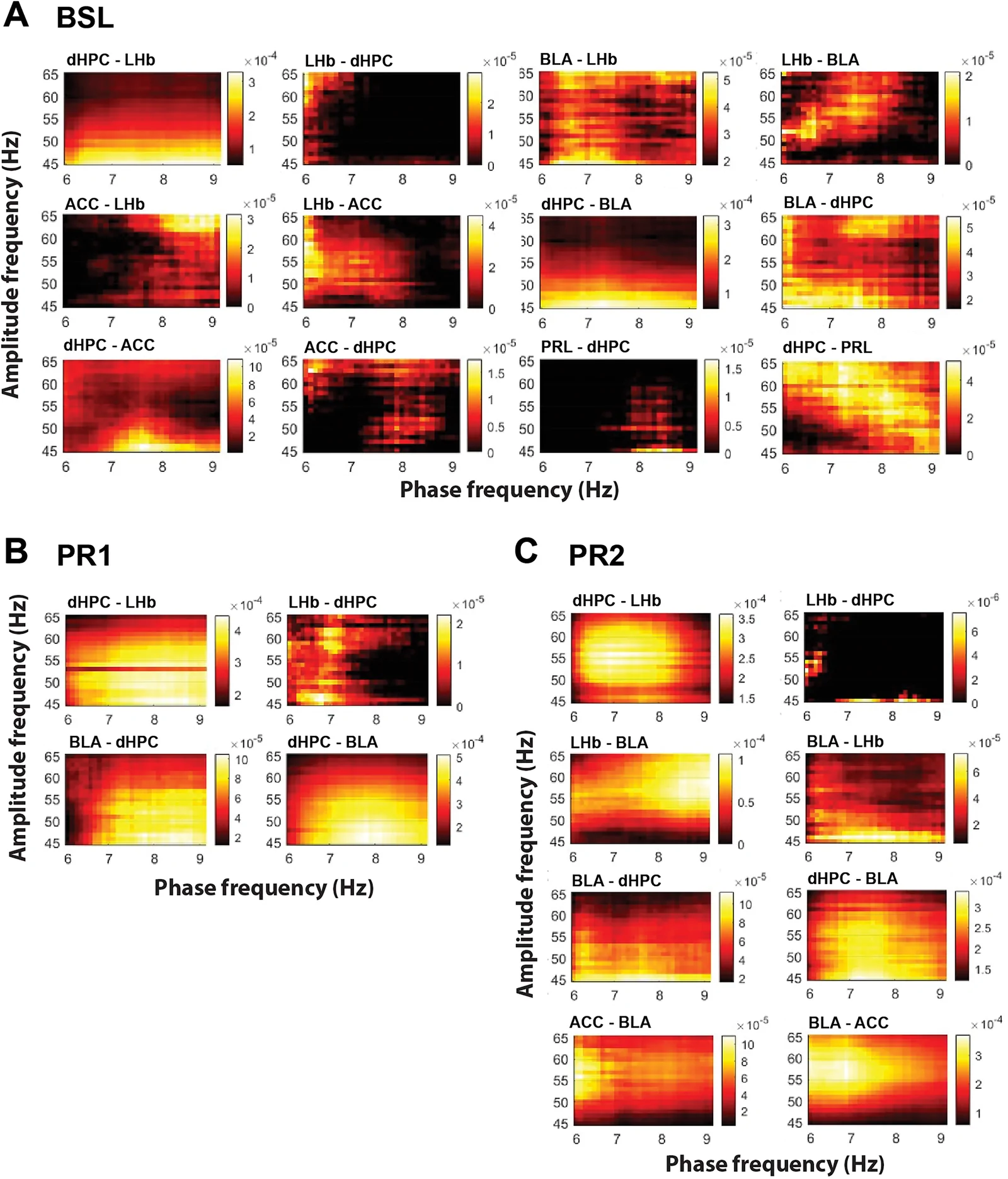
Stress and adaptation networks are associated with diverse cross-region, cross-frequency phase–amplitude coupling patterns. We calculated a modulation index, which was statistically corrected so that only those values above zero (not black) are significant. The first region listed in the title of each plot was used for theta phase and the second region was used for gamma amplitude. The *x*-axis spans a broad theta band, and the *y*-axis spans a wide gamma band, which enables visualization of changes in subregions of these two frequency bands. The panels show the modulation index between recording site pairs with significant prediction following Granger causality analysis (Fig. 4), during baseline (BSL; ***A***), post-restraint 1 (PR1; ***B***), and post-restraint 2 (PR2; ***C***). Modulation index magnitude for recording site pairs that did not have significant Granger causality is shown in Extended Data Figure 5-1.

10.1523/ENEURO.0336-25.2026.f3-5 Figure 3-5 Baseline and post-restraint behavioral features are correlated to distinct network activity. Correlation heatmaps, for both oscillatory bands [theta (upper row) and gamma (lower row)], between PCs of a given condition (BSL, PR1, PR2) and significant coherence values of the same condition. *Statistics*: bootstrap (x 1000), * indicates correlations for which the 95% confidence interval does not include zero. Download Figure 3-5, TIF file.

**Figure 6.**
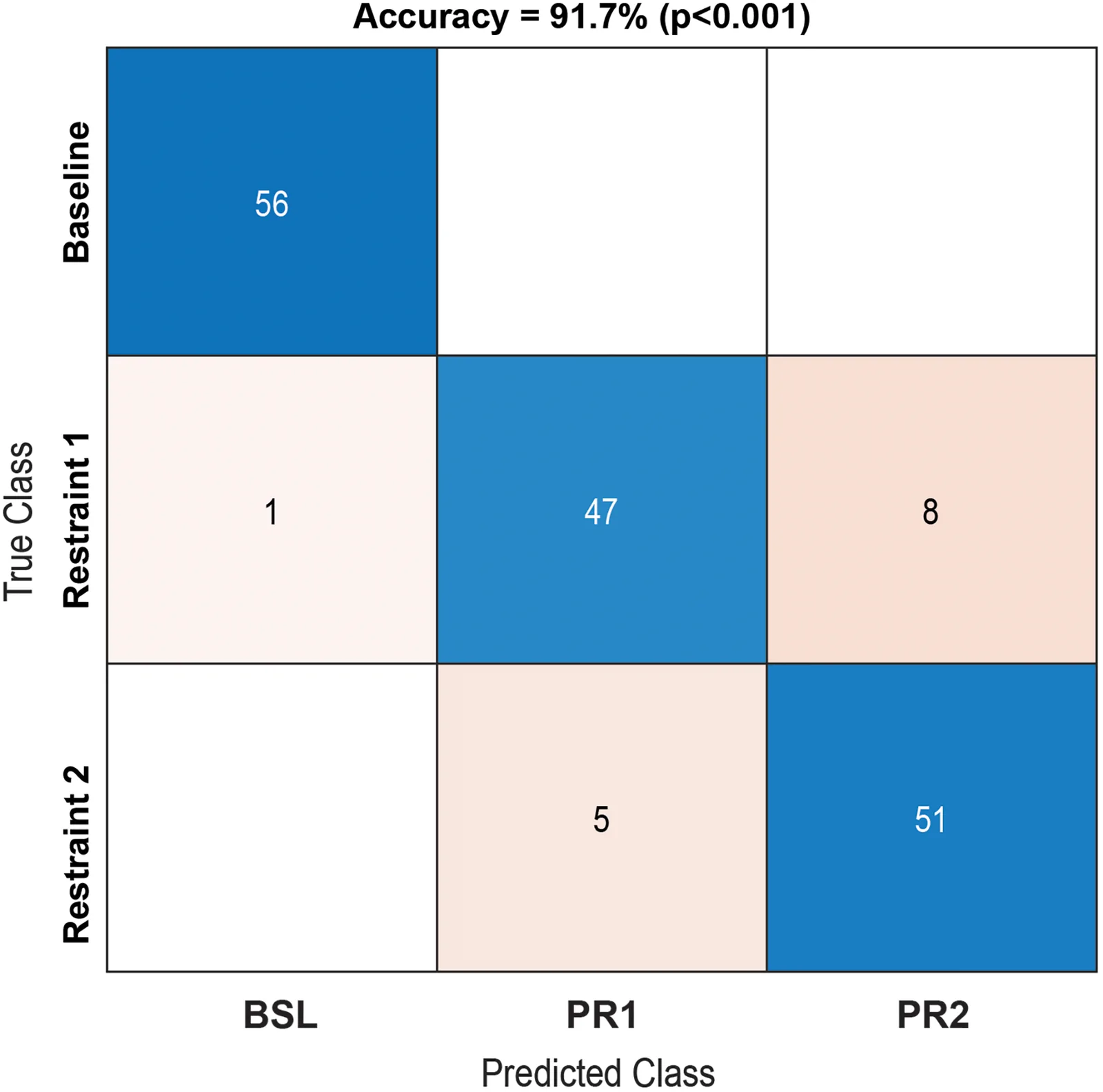
Experimental epochs can be predicted from network coherence patterns. The confusion chart shows prediction accuracy. The blue squares represent cases when the algorithm returned the correct prediction, and the red squares represent the wrong predictions. The number of observations is written in each square. Deep learning classifier settings are shown in Extended Data Figure 6-1, statistical validation and training overview of the classifier are shown in Extended Data Figure 6-2, and assessment of the generalization of the network to held-out data is shown in Extended Data Figure 6-3.

10.1523/ENEURO.0336-25.2026.f3-6 Figure 3-6 Coherence values (+ SD). Download Figure 3-6, TIF file.

### Adaptation to stress is associated with a novel network state

We have shown that the stressed state is associated with a loss of the widespread cross-region coherence observed during the pre-stress BSL. It is possible that adaptive behavior is associated with a restoration of this connectivity. We tested this hypothesis by assessing coherence during PR2. It is apparent that, in both the theta and gamma bands (Fig. 3*C*), the network activity does not return to the BSL state. There are a few notable characteristics of the network during the adaptive state. In the theta band, there is little change from PR1, other than the additional loss of ACC–PRL connectivity. In the gamma band, the network is also highly similar to PR1, except with the addition of a dHPC–BLA functional connection. Thus, stress adaptive behavior is associated with modification of the stressed (PR1) state, with both gains and losses of cross-region coherence, as opposed to a restoration of the original BSL state-network pattern.

We reasoned that the dynamics of network connectivity, in stress and adaptive states, would be correlated differentially with different behavioral expressions. Therefore, we examined the relationship between behavioral components and network activity using correlation analyses between PCA scores and significant coherence values (Extended Data Fig. 3-5). During BSL, only limited correlations were observed, i.e., a negative correlation between PC2 and hippocampocortical theta coherence. During PR1, several correlations were identified, primarily involving PC1, including negative correlations with corticocortical and cortico-BLA theta coherence, as well as ACC–PRL and cortico-BLA gamma coherence; in addition, a positive correlation was observed between PC2 and dHPC–BLA theta coherence. During PR2, correlations were no longer observed with PC1 but instead involved PC2 and PC3; specifically, PC2 showed negative correlations with BLA–ACC theta and gamma coherence and dHPC–BLA theta coherence, while PC3 was negatively correlated with BLA–ACC theta coherence. Together, these results indicate that the relationship between behavioral components and network activity differs across conditions, with distinct patterns observed between stress and adaptive states.

### Shared synaptic input does not explain network changes during stress or adaptation

The LFP signals in a pair of brain regions may be coherent due to synaptic inputs that are common to the recorded regions. Although global fluctuations in brain activity are mitigated by using the bipolar derivations of the LFP signals, as we have done above, we also calculated site-pair Granger causality magnitudes as a measure of directional causality that accounts for common synaptic input by conditioning pairwise Granger causality on all other regions in the network. Figure 4 shows that not all recording sites with significant coherence are associated with directional interactions. However, in agreement with the coherence analysis, BSL is associated with large-scale uni- and bi-directional interactions in the theta and gamma bands (Fig. 4*A*), while the stressed state (PR1) is associated with large-scale disconnection (Fig. 4*B*). In the theta band, out of the five recording site pairs with directional interactions observed during BSL, only two are spared from disconnection (dHPC–BLA and dHPC–LHb). In the gamma band, this effect is even more robust, with a total loss of network connectivity. During adaptation (PR2), the network does not return to the pre-stressed state, nor does it remain in the PR1 state (Fig. 4*C*). Instead, a gain of connectivity occurs and produces a distinct and new network state relative to the PR1 and the BSL states. In the theta band, this new network is characterized by the emergence of an ACC–BLA bidirectional connection that occurs in neither the pre-stress nor stressed states. In the gamma band, this same connection also appears during adaptation. Moreover, the total connectivity loss during PR1 is reverted with the restoration of one pre-stress pattern (LHb–BLA bidirectional interaction) and the emergence of two new patterns in the gamma band (dHPC-to-BLA and bidirectional ACC–BLA connections). Overall, these analyses suggest that a first stressful experience is associated with a general disconnection between the mPFC and related regions, and between the BLA and LHb, with the preservation of bidirectional connection between the dHPC and BLA, and dHPC to LHb connection. Adaptation to stress is associated with the restoration of the bidirectional connection between the BLA and both the ACC (theta and gamma) and LHb (theta only), as well as a novel unidirectional theta band connection between dHPC and BLA.

10.1523/ENEURO.0336-25.2026.f4-1 Figure 4-1 Granger causality values (one direction). Download Figure 4-1, TIF file.

10.1523/ENEURO.0336-25.2026.f4-2 Figure 4-2 Granger causality values (opposite direction). Download Figure 4-2, TIF file.

### Cross-frequency interactions between different regions are highly diverse

The presence of both theta and gamma band directional interactions suggests the possibility that these temporal patterns of synaptic activity may interact. Determining whether such cross-frequency interactions occur is important for generating mechanistic predictions about the cross-region drive of specific cell types or transmembrane currents that generate slower (theta band) and faster (gamma band) field potential fluctuations. We calculated the degree to which gamma band oscillation amplitude in one region had a stable pattern of temporal modulation by theta band oscillation phase in the other region (Tort et al., 2010a). Figure 5 shows the modulation index magnitude for recording site pairs that had significant site-pair Granger causality (those which did not are shown in Extended Data Fig. 5-1). Strikingly, we observed some site pairs that are largely devoid of cross-region theta phase modulation of gamma power (plots which are mostly black). Note that the lack of coupling for some site pairs shows that there is no cross-frequency interaction but does not mean that the two brain regions do not interact within a frequency band, which is shown in the Coherence and Granger causality results. Finally, for some cross-region pairs, modulations depended differently on theta phase in each region. While comparing MI values in a repeated-measures ANOVA is not possible (because MI values that are not indicative of any cross-frequency coupling at all will still have a value), Figure 5 shows the diversity of cross-region, cross-frequency interactions in the stress and adaptation networks. Our results clearly show that cross-frequency network interactions occur in the context of stress and adaptation (something not possible to demonstrate using postmortem cFos) and, furthermore, that the network patterns qualitatively differ between stressed and adaptive states.

10.1523/ENEURO.0336-25.2026.f5-1 Figure 5-1 Theta-Gamma phase-amplitude coupling patterns during baseline (BSL; **A**), post-restraint 1 (PR1; **B**), and post-restraint 2 (PR2; **C**). Here are presented coupling in pairs that did not show significant prediction following Granger causality analysis (**Fig. 4**). Download Figure 5-1, TIF file.

### Stress and adaptive states can be decoded from network activity

We observed distinct neuronal network activity associated with pre-stress, stressed, and adaptive states. We sought to decode the pre-stress, stressed, and adaptive states from the coherence magnitudes recorded across the network. We chose coherence, over Granger causality, because coherence can be calculated online with low latency, which may have translational potential. We trained a feedforward neural network and tested it on held-out data. The experimental epochs (BSL, PR1, PR2) could be predicted from network coherence patterns with an average accuracy of 91.7% (Fig. 6, *p* < 0.001).

10.1523/ENEURO.0336-25.2026.f6-1 Figure 6-1 Deep learning classifier settings. Download Figure 6-1, TIF file.

10.1523/ENEURO.0336-25.2026.f6-2 Figure 6-2 Statistical validation and training overview of the classifier over the classes (BSL, PR1, PR2). The classes associated with each observation were shuffled (1000 repetitions) to assess that the results obtained were not spurious. We obtained a bootstrap distribution around 0.33, which corresponds to the fact that there were 3 classes. (**A**) Distribution of the bootstrap made on randomly relabeled observation (x 1000). The red line shows the location of the experimental observation. (**B**) Cross–Entropy Loss over the number of iterations during training of the classifier of the original observations representing how well the classifier performs. Download Figure 6-2, TIF file.

10.1523/ENEURO.0336-25.2026.f6-3 Figure 6-3 Assessment of the generalization of the network to held out data. (**A**) Confusion chart of Test 1 of the neuronal network classifier based on 9 rats, which achieved accuracy of 93.3 %. (**B**) Confusion chart of Test 2, on the held-out rat. The accuracy was 100 % correct. Download Figure 6-3, TIF file.

Prediction was also successfully achieved when nine out of the 10 rats were used for training and the prediction made with data from the remaining rat (Extended Data Fig. 6-2). Therefore, coherence across this network holds translational potential for tracking stress and adaptation.

## Discussion

We aimed to characterize functional connectivity associated with stressed and adaptive states among a cerebral network of stress-responsive brain regions, including the PRL, ACC, BLA, LHb, and dHPC (Jacinto et al., 2013; Durieux et al., 2020, 2022; Merino et al., 2021; Marques et al., 2022). We performed two consecutive restraints and simultaneously recorded LFPs while the behavioral responses during and consecutively to restraint were scored. We found that there was a behavioral response to the first stressor and a return to baseline after the second stressor. In our opinion, this represents a behavioral indicator of adaptation to such a stressful event, as the response resembles that initiated in its absence, suggesting rats overcome stress exposure. This is reminiscent of findings resulting from rodent paradigms such as chronic stress exposure, to which some individuals show resilience, i.e., they display behavioral responses similar to nonstressed animals, while others that are vulnerable to stress exposure show exacerbated behavioral responses, which was not the case here. We report that distinct neuronal network states are associated with baseline, stressed, and adaptive states. The baseline state was characterized by robust global connectivity, the stressed state by sparse connectivity, resulting in a novel connectivity pattern during the adaptive state compared with the pre-stress baseline. In addition, PCAs indicate that although the magnitude of the behavioral response is similar between BSL and PR2, there are distinct contributions of the different behaviors, strengthening the view that both epochs can be defined by different behavioral profiles and strengthening the view that PR2 can be regarded as adaptation. Finally, we show that experimental epochs can be decoded from the network state. In interpreting our results, it is important to note that the LFP is dependent upon the orientation of the surrounding neurons, and the cytoarchitecture of some subcortical regions poses difficulty in interpreting the LFP. Nevertheless, these recordings show the dynamics of synaptic inputs coordinated across a network of forebrain regions, which has heretofore relied on indirect measures of neuronal metabolism and neuronal activation garnered from slow, hemodynamic signals.

### Pre-stress baseline is associated with widespread network connectivity

While it is known that the PFC, HPC, and AMY have coordinated activity in awake rats (Jacinto et al., 2013; Stubbendorff et al., 2023), our findings suggest that the LHb is also engaged in this forebrain network. For instance, we observed LHb–dHPC theta coherence. This pattern has been observed in awake rats and in the context of cognitive functions (Aizawa et al., 2013; Goutagny et al., 2013). We also observed coherence between the LHb and the BLA, as well as the mPFC, in line with prior studies using Fos, a static indirect measure (Durieux et al., 2020, 2022). Overall, the present findings indicate widespread forebrain connectivity, including, as shown for the first time, the LHb.

### Stress produces widespread network disconnection

Animal studies of the neurophysiological consequences of stress exposure have been primarily in the domain of early life stress or chronic stress, and there is paucity of functional connectivity data concerning acute stress and subsequently adaptation with repetitions of the stressor. Decreased BLA–mPFC theta coherence has been reported in rats after maternal separation (Cao et al., 2016) and after chronic exposure to an elevated platform (Lee et al., 2011). We observed no change. Gamma coherence has been shown to increase between dHPC and PRL/ACC as well as between ACC and PRL after chronic social defeat stress. We observed no change in this aspect of the network either. Therefore, we conclude that the brain state evoked by the acute stress paradigm used in the present study may differ from the brain state formed during chronic stress. Our study involves regions related to the attention/cognitive control and threat/salience circuitries, as proposed by Nestler and Russo (2024), and implicates specifically the theta and gamma bands in the neuronal network state during acute stress. This finding is in line with prior work on how neuronal activity is coordinated between two brain regions during acute stress (Colgin, 2013; Headley and Paré, 2013). For instance, during fear conditioning, the AMY and HPC, as well as the AMY and PFC, have coherent LFP signals in these bands (Seidenbecher et al., 2003; Likhtik et al., 2014). Moreover, similar findings have been reported between the PFC and HPC in an anxiogenic context (Adhikari et al., 2010).

### Neuronal network activity may have translational potential for assessing stressed and adaptive states

We observed that the stressed state was associated with reduced network connectivity, whereas the adaptive state was characterized by a reorganization of connectivity patterns. These network states were compared with a pre-stress baseline (which itself had robust connectivity, but in a different pattern than the adaptive state). However, it is possible that the state following the second stressor was a transition state and that, with adequate time, the network might relax into a baseline-like state. Similarly, the baseline state network configuration may itself be dynamic. Future work can address these questions using multiday pre-stress baseline recordings and continuous (24 h per day) recordings after the second stressor. If the pre-stress, stressed, and adaptive states are associated with stable forebrain networks, then our findings hold translational potential for understanding stress and adaptation. Consistent with this potential, we were able to successfully predict with high accuracy (>90%) the experimental epochs from the forebrain network activity. Translational measures of stress and adaptation have typically used peripheral measures that correlate with activity of the autonomic nervous system (Carnevali and Sgoifo, 2014) or circulating corticosterone (Armario et al., 2023). Our results suggest that monitoring field potentials may hold promise for tracking stress and adaptation. Neuromodulation therapies aimed at steering neuronal networks toward a state of adaptation may also need to consider that adaptation corresponds to a distinct brain state, rather than a simple return to the pre-stress baseline

There are nonetheless some limitations to this study. One is that the stressors occurred on a different day than the baseline to which they were being compared. A second limitation is that we did not analyze the dynamics of the stress response (behavioral or neural) continuously throughout the experiment and instead focused on 1 min windows proximal to the stress; however, since it is excessively challenging, behaviorally, to know which time points best capture the primary stress response, we chose to focus on one time point at which we could be certain that the rat was processing a recent stress experience. Although one strength of this approach is that we applied local referencing to reduce volume conduction, which is critical for recording nearby brain regions, while this manipulation actually improves the accuracy of the Granger Causality calculation (Trongnetrpunya et al., 2016), a downside is that it reduces power; however, referencing was applied uniformly across the task epochs being compared, so any reduction in power was taken into account. Nevertheless, it is important to note that comparing the absolute power or coherence values between the present study and other studies may show differences that are due to the presence of bipolar referencing. Finally, there are known sex differences in stress responses and prevalence of stress-related psychiatric disorders; as here we only used male rats, future experiments are needed to repeat this work treating sex as a biological variable

In summary, we observed specific patterns of neuronal network activity between regions in a cortical-hippocampal-amygdalar-habenular network upon repeated exposure to a stressor, with adaptation giving rise to a new network state that resembles neither the baseline nor the stressed state. This view is strengthened by the outcome of the correlational analysis between behavioral PCA scores and coherence values, showing distinct patterns of correlation across all epochs. Previous studies have demonstrated that the expression of emotional responses such as stress, fear, or anxiety was accompanied by increased interregional connectivity, between for example the HPC and the AMY (Seidenbecher et al., 2003), the mPFC and the AMY (Stujenske et al., 2014; Hultman et al., 2016), or the infralimbic cortex and the thalamus (Carlson et al., 2017); interestingly in the later case the authors found that upon repetition of the stressor (two-tail suspension sessions in mice, 1 d apart), cellular activity was increased in both regions and their coupling augmented, during the second exposure. Our study extends these findings by examining, in the same animals, the evolution of network activity across repeated stress exposure, including both connectivity and directionality of information transfer. We show that distinct patterns of network activity emerged between stressed and adaptive states. We show that both brain states can also be accompanied by loss of connectivity. It likely depends on the protocol used and the regions explored, but it nonetheless proposes that opposite dynamics, gain and loss of connectivity at frequency bands such as theta and gamma, possibly in relation to various types of information processed, can concomitantly occur within specific networks in order to properly respond to repeated stressor exposure. Finally, these findings suggest that treating stress-related disease may require modulating neuronal networks into a new state, rather than “normalizing” the brain to its original state prior to stress. The ability to predict pre-stress, stress, and adaptive states from the neuronal network state holds potential translational usefulness in tracking and treating stress-related diseases.

## Synthesis

Reviewing Editor: Katrina Choe, McMaster University Decisions are customarily a result of the Reviewing Editor and the peer reviewers coming together and discussing their recommendations until a consensus is reached. When revisions are invited, a fact-based synthesis statement explaining their decision and outlining what is needed to prepare a revision will be listed below. The following reviewer(s) agreed to reveal their identity: Tamas Fuzesi.

The reviewers commented that the revised manuscript adequately addressed previously raised issues, except for a couple of remaining concerns:

1. The summary behavior data (Figure 2 panel B) should be removed if a multiple comparison test is not applied in the first section of Figure 2.

2. The definition and interpretation of "adaptation" should be better clarified.

## Author Response

Dear Dr Choe, We thank you very much for this reviewing process, which undoubtedly led to the great improvement of our manuscript.

According to the remarks, we have:

1) deleted Figure 2B and corrected the legend of Figure 2, as well as the result section, accordingly;

2) tried to better define what we meant by "adaptation" (first paragraph of the introduction, highlighted in yellow), and to bring some argument in the discussion, following mention of the behavioral responses observed, to compare what we obtained to what is obtained for example in paradigms such as chronic stress exposure (highlighted in yellow).

According to the uploaded files, we have only replaced the two manuscript files (article file and clean copy), with the mention of "R2" at the end of the file's names, and replaced Figure 2 with the corrected one (also with "R2" at the end of the file's name).

We do hope that it will be ok.

With our Best Regards, The authors.
